# Astrocytes in Neural Circuits: Key Factors in Synaptic Regulation and Potential Targets for Neurodevelopmental Disorders

**DOI:** 10.3389/fnmol.2021.729273

**Published:** 2021-09-29

**Authors:** Xing Liu, Jun Ying, Xifeng Wang, Qingcui Zheng, Tiancheng Zhao, Sungtae Yoon, Wen Yu, Danying Yang, Yang Fang, Fuzhou Hua

**Affiliations:** ^1^Department of Anesthesiology, The Second Affiliated Hospital of Nanchang University, Nanchang, China; ^2^Key Laboratory of Anesthesiology of Jiangxi Province, Nanchang, China; ^3^Department of Anesthesiology, The First Affiliated Hospital of Nanchang University, Nanchang, China; ^4^Mailman School of Public Health, Columbia University, New York, NY, United States; ^5^Helping Minds International Charitable Foundation, New York, NY, United States

**Keywords:** synaptic plasticity, astrocyte, glial transmitter, D-serine, glutamate, astrocyte-neuron signaling

## Abstract

Astrocytes are the major glial cells in the brain, which play a supporting role in the energy and nutritional supply of neurons. They were initially regarded as passive space-filling cells, but the latest progress in the study of the development and function of astrocytes highlights their active roles in regulating synaptic transmission, formation, and plasticity. In the concept of “tripartite synapse,” the bidirectional influence between astrocytes and neurons, in addition to their steady-state and supporting function, suggests that any negative changes in the structure or function of astrocytes will affect the activity of neurons, leading to neurodevelopmental disorders. The role of astrocytes in the pathophysiology of various neurological and psychiatric disorders caused by synaptic defects is increasingly appreciated. Understanding the roles of astrocytes in regulating synaptic development and the plasticity of neural circuits could help provide new treatments for these diseases.

## Introduction

Astrocytes play an important role in terms of both function and their abundance in the central nervous system. For many years, astrocytes have been considered to play a supporting role in neural functions, such as ion homeostasis, regulation of local blood flow, clearance of neurotransmitters, and energy supply of neurons. In recent years, it has been found that astrocytes are also functional elements of “tripartite synapses” with presynaptic and postsynaptic neurons (Araque et al., 1999). Astrocytes control the formation, maturation, and plasticity of synapses through a variety of secretory and contact-mediated signals.

There are highly complex neural networks in the mammalian nervous system. Since a single neuron rarely performs a function on its own, it must establish connections with other neurons and form neural circuits through synapses. Therefore, the correct synaptic connection is the key to the formation of functional neural circuits. With the continuous production of immature synapses during development, some synapses with repeated inputs will become mature or stable, while other excess synapses will be eliminated or pruned (Clarke and Barres, 2013). Precise neural circuits are formed by initially overproducing neurons and synapses, followed by the elimination or pruning of superfluous neurons and synapses.

Synaptic plasticity refers to the enhancement or weakening of long-term synaptic transmission related to activity, which is typically achieved through long-term potentiation (LTP) and long-term depression (LTD) (Yong et al., 2020). Synaptic remodeling occurs throughout life in diverse areas, prominently including the hippocampus, which is a structure essential for learning and memory consolidation (Nguyen et al., 2020). Synaptic morphological changes are associated with various neurodevelopmental disorders, including schizophrenia, autism spectrum disorders, and neurodegenerative diseases such as Alzheimer's disease and Parkinson's disease (Faludi and Mirnics, 2011; Li et al., 2016). Astrocytes participate in some pathophysiological processes of these diseases through the regulation of synapses and other effects. This review discusses the influence of astrocytes on the development of dendritic protrusions and synaptic plasticity during neural circuit development, while further deepening the understanding of the link between this influence and human neurodevelopmental disorders.

## The Calcium Signal of Astrocytes Participates in the Transmission of Information Between Astrocytes and Neurons and Affects Neural Activity

In contrast with neurons, which can produce action potentials, astrocytes are electrically non-excitable cells. Instead, theyG-protein-coupled metabolic receptors such as mevel to change the response of neurons (Cornell-Bell et al., 1990; Pasti et al., 1997). Therefore, Ca2+ signals are commonly measured as an indicator of astrocyte reactivity.

Research data shows that G protein-coupled metabolic receptors, such as metabotropic glutamate receptors, type 1 cannabinoid (CB1) receptors, purinergic receptors, acetylcholine receptor (AchR), and GABAb receptors, or ligand-gated ion channel NMDA receptors (Kang et al., 1998; Papouin et al., 2017), are activated by corresponding physiological ligands. Hippocampal astrocytes in the region of the oriens layer of CA1 receive large amounts of cholinergic input and respond to acetylcholine released by synaptic terminals. The synaptically released ACh acts on mAChRs, causing the release of internally stored Ca^2+^ (Araque et al., 2002). Indeed, astrocyte-neuron communication is complex. By using Ca^2+^ imaging and electrophysiology technology in hippocampal slices of mice, researchers found that Astrocytes can distinguish between cholinergic and glutamatergic synaptic activity and also between synaptic activity belonging to different axon pathways. Because glutamate released from alveus axons does not cause elevated Ca^2+^ levels as glutamate released from Schaffer collaterals and Ach released from alveus axons (Araque et al., 2002; Perea and Araque, 2005). This undoubtedly highlights the ability of astrocytes to integrate synaptic information in the astrocyte-neuron communication.

Additionally, Phospholipase C can be activated to produce inositol IP3, which subsequently binds to specific receptors and induces the release of Ca^2+^ from the endoplasmic reticulum into the cytoplasm. The level of intracellular Ca^2+^ increases significantly, and a corresponding signal is transmitted in the form of Ca^2+^ waves. IP3-induced Ca^2+^ release is essential for initiating and maintaining Ca^2+^ waves between neurons and astrocytes. Most metabolic neurotransmitter receptors expressed in astrocytes activate phospholipase C/IP3 signal cascades. However, because the expression of IP3 type 2 receptor (IP3R2) in astrocytes is higher than in neurons, it leads to the depletion of stored Ca^2+^. The significant increase of intracellular Ca^2+^ is mainly induced by a combination of IP3 and IP3R2 in the endoplasmic reticulum (Hamada and Mikoshiba, 2020). This pathway is considered to be the basis of astrocytic Ca^2+^ waves, which are relatively slow calcium signals.

At the same time, the release of Ca^2+^ is amplified by the recruitment of the calcium-dependent endoplasmic reticulum Ca^2+^ channel, IP3 receptor (IP3s), and ryanodine receptor (RyR), which further lead to the increase of Ca^2+^ levels. Therefore, this is considered the main pathway that activates the calcium signal of astrocytes in the brain.

In addition to the fact that relatively slow Ca2+ waves can affect neuronal activity through astrocyte responses, transient changes of intracellular Ca2+ concentration ([Ca2+]i) in astrocytes can also regulate neuronal activity by inducing astrocytes to produce different responses (Araque et al., 2014). However, the reason why this transient [Ca2+]i is not regarded as the starting point for the activation of calcium signals in astrocytes in the brain is that the transient [Ca2+]i of astrocytes can be blocked without blocking neuronal transient [Ca2+]i or neuronal synaptic activity by blocking the above-mentioned mGluRs and other different receptors by using specific receptor antagonists. In addition to these intracellular signaling pathways, it has been shown that the release of stored Ca^2+^ in cells downstream of G-protein-coupled metabolic receptors (also including other receptors) induces transient [Ca^2+^]_i_ in astrocytes. However, recent studies have challenged this concept because the cellular processes of astrocytes adjacent to neuronal synapses lack Ca^2+^ storage (Patrushev et al., 2013), which means that transient [Ca^2+^]_i_ cannot be produced through intracellular storage of Ca^2+^ in these astrocytes to affect the release of synaptic transmitters. Moreover, when IP3R2 was knocked out, the number of astrocytes with transient [Ca^2+^]_i_ was greatly reduced, but the role of astrocytes in neuronal excitability (Petravicz et al., 2008), synaptic electrophysiology (Petravicz et al., 2008), and synaptic plasticity (Agulhon et al., 2010) was not affected. These studies show that the previous view that the effects of glial transmitters released by astrocytes on neuronal function are all caused by IP3-induced calcium release from the endoplasmic reticulum of astrocytes caused by transient [Ca^2+^]_i_ needs to be further studied.

Indeed, different astrocyte processes produce transient [Ca2+]i at various times (Nett et al., 2002), and astrocyte processes with spatially localized transient [Ca2+]i occur much more frequently than in somatic cells (Grosche et al., 1999; Nimmerjahn et al., 2009; Kanemaru et al., 2014). Furthermore, the advent of new techniques such as two-photon fluorescence imaging has made it easier to characterize different types of calcium transients in different parts of astrocytes. Transient [Ca2+]i in astrocyte processes can spread along the process into the soma (Nett et al., 2002; Kanemaru et al., 2014), and this signal transmission also occurs between cells (Nimmerjahn et al., 2009). The investigators introduced the Ca2+ chelator BAPTA into astrocytes in the dentate gyrus (Castro et al., 2011) and hippocampal area CA1 (Panatier et al., 2011) regio, causing an increase in the rate of synaptic failure. These studies invariably illustrate that increases of [Ca2+]i in astrocytes are essential for the regulation of neuronal and synaptic activity.

What's more, a recent study showed that although some transient [Ca2+]i astrocyte of were eliminated when IP3R2 was knocked out, this loss of store-release receptors did not have the anticipated major effect on astrocytes (Kanemaru et al., 2014; Srinivasan et al., 2015). In fact, a release of Ca2+ from internal stores is the main source of somatic transient [Ca2+]i, but the transmembrane entry of Ca2+ in astrocytic processes, which may be mediated by endogenously active channels such as TRPA191 or receptor-gated Ca2+-permeable ion channels, produces a 30–40% elevation of [Ca2+]i (Srinivasan et al., 2015). Therefore, Ca2+ signaling in astrocytes differs greatly between the soma and processes. Moreover, there are at least 8 times more transient [Ca2+]i in the processes than in the somata (Kanemaru et al., 2014; Srinivasan et al., 2015). Thus, previous conclusions based on knockdown of IP3R2 receptors actually did not adequately take into account the difference in Ca2+ responses between somatic cells and astrocytes, but the modulatory effects of astrocytes may be critical.

Some researchers have also proposed other pathways for the increase of [Ca^2+^]_i_ in astrocytes. For example, extracellular Ca^2+^ can flow into the cytoplasm due to the activation of Ca^2+^ permeable glutamate channels, which are composed of NMDARs and AMPA receptors (AMPARs) (Steinhäuser and Gallo, 1996). One study showed that Ca^2+^ enters mature hippocampal astrocytes mainly through NMDARs. Extracellular Ca^2+^ can also enter astrocytes through voltage-gated calcium channels (VGCCs), and the expression of VGCCs seems to decrease with age (Steinhäuser and Gallo, 1996). The activation of transient receptor potential C-type channels (TRPCs) can also induce the entry of extracellular Ca^2+^ into astrocytes. As a non-selective calcium-permeable cation channel, TRPC can not only be activated by the production of IP3 and depletion of stored Ca^2+^ (Birnbaumer, 2009), but also participates in the increase of intracellular Ca^2+^ through store-operated Ca^2+^entry independent of the PLC/IP3 signal cascade (Schwarz et al., 2019).

It is certain that the Ca^2+^ signal can regulate the release of glial transmitters in astrocytes. However, there must be other determinants of their release, such as the subcellular localization of receptors relative to internal calcium storage.

**Figure 1:**
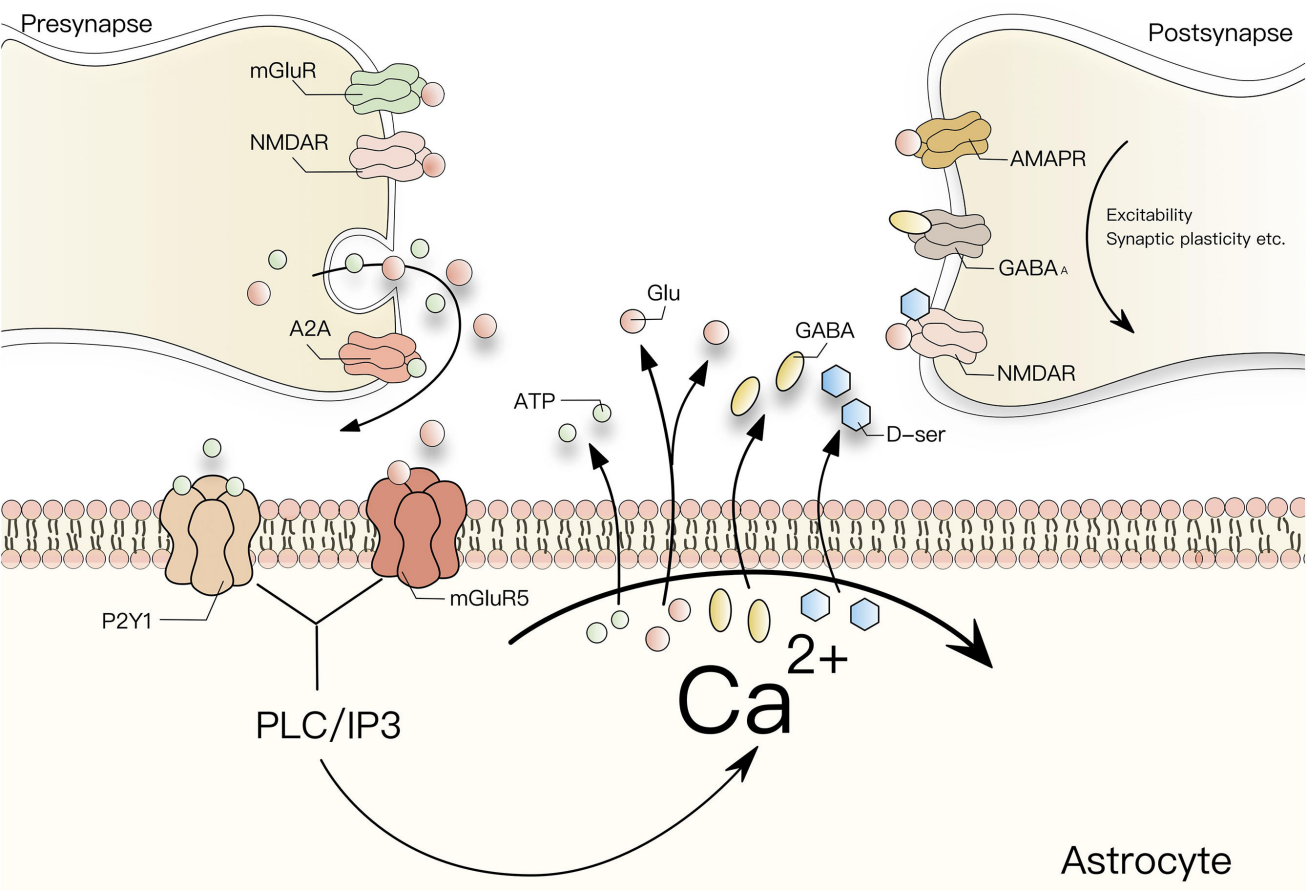
Interaction between Ca^2+^ and glial transmitters. G-protein-coupled receptors activated by glutamate, GABA, and ATP can increase [Ca^2+^]_i_ in astrocytes through the PLC/IP3 signal cascade. The increase of [Ca^2+^]_i_ can in turn enhance the release of glial transmitters such as glutamate, GABA, ATP or D-ser by astrocytes and influence the excitability, synaptic plasticity, and synaptic electrophysiology of postsynaptic neurons. Among them, glutamate induces an NMDAR-mediated inward membrane current in neurons to regulate synaptic excitability and synchronize its action potential. ATP mainly activates the P2y1 receptor from the P2 receptor family, which increases [Ca2+]i, and the release of D-ser also plays a similar role by binding with NMDAR. And astrocytes increase the transmission efficiency of CA1 pyramidal cells by activating the subtype 5 metabotropic glutamate receptor (mGluR5) and presynaptic adenosine A2A receptor, resulting in the upregulation of synaptic transmission and induction of LTP At the same time, D-ser controls the activation of NMDA receptors when glutamate is released from synapses.

As mentioned earlier, Ca^2+^ is closely involved in the interconnection between neurons and astrocytes. Astrocytes can affect neuronal excitability, neurovascular coupling, synaptic electrophysiology, and synaptic plasticity by regulating [Ca^2+^]_i_. The effects of these signals on synaptic plasticity are not explained in detail here. At present, it is believed that Ca^2+^ has a prominent remodeling effect on neurons mainly by affecting the release of glial transmitters from astrocytes. According to previous studies, different receptor agonists such as glutamate and GABA can increase [Ca^2+^]_i_ in astrocytes and induce astrocytes to release glial transmitters, including glutamate, GABA, D-serine (D-ser), ATP, and prostaglandins. The relationship between Ca^2+^ and glial transmitter release is summarized in Figure 1. The release of GABA, glutamate, and ATP can increase [Ca^2+^]_i_ by activating presynaptic receptors, whereby ATP mainly activates presynaptic P2Y1 receptors from the P2 receptor family, while glutamate induces NMDAR-mediated currents to regulate the release of synaptic vesicles to affect synaptic strength and regulate synaptic plasticity (Fattorini et al., 2019). In addition, Ca^2+^ waves also mediate the synaptic plasticity of toxic acetylcholine receptors in the somatosensory cortex and play a role in synaptic structural integrity, which depends on IP3R2 signals and extracellular D-ser (Norio et al., 2011). In a mouse model lacking the IP3 signal, the coverage of astrocytes in asymmetric synapses decreased and led to changes in the release of glutamatergic transmitters (Mariotti et al., 2015). In conclusion, the [Ca2+]i transient [Ca^2+^]_i_ of astrocytes is essential both for the regulation of neuronal activity and synaptic plasticity.

## Roles of Astrocytic Factors in the Regulation of Excitatory Synaptic Function

### Astrocytic1 Neurotransmitters

Synaptic plasticity has many manifestations. The typical types of synaptic plasticity include LTP and LTD. Studies have shown that astrocytes can regulate synaptic transmission and participate in the regulation of excitatory synaptic structure and function by releasing glial transmitters such as glutamate and D-ser in response to Ca^2+^ influx (Araque et al., 2014).

#### Glutamate

There are multiple glutamate release mechanisms in astrocytes. Under physiological conditions, glutamate is mainly released from astrocytes by exocytosis. Other Ca^2+^-release channels, such as Ca^2+^-dependent semi-channels, anion transporter channels, and P2X receptor channels are mostly closed under physiological conditions, and are mainly related to pathological states such as epilepsy and injury (Vardjan et al., 2017).

As mentioned earlier, the neurotransmitters and other signals released by the synapses of neurons increase the level of intracellular Ca^2+^, which in turn activates exocytosis of glutamate by astrocytes and also activates glutamate receptors on the synaptic membrane. For example, glutamate exocytosis can activate presynaptic NMDA receptors (NMDARs) to increase Ca^2+^ levels and the excitatory current, thereby enhancing excitatory synaptic transmission in hippocampal dentate granule cells (Jourdain et al., 2007). In other words, this type of LTP is caused by the activation of NMDARs. In addition, NMDAR-dependent LTD or LTP can also be affected by the increase or decrease of AMPAR abundance in the postsynaptic membrane.

The activation of group I metabotropic glutamate receptors is reflected in the decrease of postsynaptic AMPAR abundance, while LTD is induced when the number of AMPAR increases (Skowrońska et al., 2019). Although the number of postsynaptic AMPAR is always changing, synaptic transport through AMPAR can achieve dynamic balance, which is a complex process involving extracellular signaling circulation, and lateral diffusion (Choquet, 2018). More specifically, AMPAR is mobility as synaptic receptors, and both for extracellular circulation and lateral diffusion, reveal a continuous high mobility exchange of AMPAR between synaptic and extrasynaptic membranes.The extrasynaptic membrane of hippocampal pyramidal neurons contains a large number of AMPARs, which can be used as reserve materials, and dynamically exchange between synaptic and extra-synaptic sites through lateral diffusion (Tao-Cheng et al., 2011). Also, AMPAR can circulate between the intracellular pool and the cell surface pool through exocytosis or endocytosis, and through extracellular circulation to control its dynamic balance. This balance can be changed according to the needs of neuronal activity (Opazo and Choquet, 2011). For example, in the process of LTP, AMPAR can increase the abundance of postsynaptic AMPAR through lateral diffusion, and correspondingly, AMPAR excreted to maintain the dynamic balance naturally acts as an extra-synaptic reservoir (Makino and Malinow, 2009; Penn et al., 2017). Conversely, during LTD, the endocytosis of AMPAR reduces its activity from the postsynaptic membrane to reduce synaptic activity. In addition, the release of glutamate can trigger another form of LTP that does not depend on postsynaptic NMDAR-mediated signals but requires presynaptic mGluR activation (Perea and Araque, 2007).

As the third subfamily of iGluRs, the kainate receptors (KARs) have received much less attention. There are five members in the KARs family, GluK1 to GluK5, all with different functions. GluK2 and GluK5 are mainly found in neurons. GluK3 is expressed in granule cells of the dentate gyrus, while GluK1 is widely expressed during the entire whole process of growth and development but is confined to hippocampal intermediate neurons after full development (Lerma et al., 2001; Pinheiro et al., 2007; Vesikansa et al., 2007). In contrast to AMPARs, KAR-mediated currents are small in amplitude with slow activation and deactivation kinetics (Castillo et al., 1997; Frerking et al., 1998; Vignes et al., 1998). KARs are slowly activated or inactivated by Neto protein, an auxiliary subunit of KARs (Lerma, 2011; Jon et al., 2016) which gives KARs a variety of abilities to regulate synaptic plasticity (Straub et al., 2011; Sylwestrak and Ghosh, 2012; Lerma and Marques, 2013). Just like other iGluRs, KARs can be activated by Go protein, phospholipase C (PLC) and protein kinase C (PKC) metabolic cascade, but the mechanism of KAR activation in this atypical metabolic pathway has not been well-explained (Dubois et al., 2016; Duan et al., 2018; Tao et al., 2019). KARs are widely distributed in the presynaptic area (Schmitz et al., 2001; Lerma, 2006), and, together with multiplicity of signaling mechanisms of KARs, this may be a powerful factor affecting the regulation of presynaptic plasticity.

In addition to the iGluRs, the effects of astrocytic glutamate on mGluRs are also significant for the regulation of synaptic plasticity. In fact, mGluRs are extensively involved in the regulation of neuronal excitability and synaptic transmission throughout the CNS, as glutamate can regulate neuronal cell excitability as well as synaptic transmission through the second messenger signaling pathway (Sladeczek et al., 1985; Sugiyama et al., 1987). Metabotropic glutamate receptors (mGluRs) are differentially expressed in various cell types throughout the CNS, and to date, eight subtypes of mGluRs (Sladeczek et al., 1985; Sugiyama et al., 1987) have been characterized and classified into three subgroups based on sequence homology and cellular signaling activation (Gerber et al., 2007). The first group includes mGluR1 and mGluR5, the second group includes mGluR2 and mGluR3, while the third group includes mGluR4,6,7,8. The mGluRs are widely distributed in neurons, except for mGluR4, which is mainly distributed in the retina (Table 1).

**Table 1:** Distribution of mGluR in CNS.

**Group**	**Receptor**	**CNS distribution**	**References**
Group I	*mGluR1*	Widespread in neurons (cerebellar olfactory tubercle, cerebral cortex, dentate gyrus, lateral septal nucleus, striatum, nucleus accumbens, amygdaloid nuclei, substantia nigra pars reticulata and cerebellar cortex)	Pin and Duvoisin, 1995; Kniazeff et al., 2011; Ribeiro et al., 2011
	mGluR5	Widespread in neurons (cerebral, cortex, Hippocampus, subiculum, olfactory bulb, striatum nucleus accumbens lateral septal nucleus)	Abe et al., 1992; Shigemoto et al., 1993; Romano et al., 1995
		Astrocytes	Biber et al., 2010; Schools and Kimelberg, 2015
Group II	mGluR2	Widespread in neurons (cerebellar cortex and olfactory bulb)	Ohishi et al., 1993, 1994
	mGluR3	Widespread in neurons (cerebellar olfactory tubercle, cerebral cortex, dentate gyrus, lateral septal nucleus, striatum, nucleus accumbens, amygdaloid nuclei, substantia nigra pars reticulata and cerebellar cortex)	Tanabe et al., 1993; Testa et al., 1994; Petralia et al., 1996; Ohishi et al., 2010
		Astrocyte	Tanabe et al., 1993; Ohishi et al., 1994, 2010
Group III	mGluR4	most intense in the cerebellum	Fotuhi et al., 1994; Kinoshita et al., 1996; Makoff et al., 1996; Azkue et al., 2015
	mGluR6	Retina	Nakajima et al., 1993
	mGluR7	Widespread in neurons	Kinoshita et al., 2015
	mGluR8	Lower and more restricted than mGluR4/7	Duvoisin et al., 1995; Saugstad et al., 1997; Corti et al., 1998

The iGluRs and the mGluRs are constantly regulated in both directions, and group I mGluR activators enhance the activation of NMDAR-containing GluN2A/2B within minutes (Fitzjohna et al., 1996; Lan et al., 2001; Benquet et al., 2002). Interestingly, this effect appears to be a cell context specific, as enhancement of NMDAR occurs primarily in hippocampal neurons and NMDAR is inhibited in cortical neurons. Similar findings were reported for group I mGluR activation leading to the enhancement in CA1 neurons and inhibition of NMDAR currents in CA3 neurons (Grishin, 2004).

Group I mGluRs can also regulate other iGluRs. Internalization of receptors as a classical form of regulation leads to a reduction in the abundance of membrane receptors and downregulation of their function. In the context of studies on synaptic plasticity, the main focus has been on AMPAR, and group I mGluRs can lead to their internalization through a variety of kinases and phosphatases (Moult, 2006). In fact, not only AMPAR is affected by mGluR-induced internalization of iGluRs, since the number of NMDARs also decreases in response to mGluR activation (Snyder et al., 2001). From the aspect of kainic acid, mGluR in group I mainly enhance containing GluK5 through PKC-mediated phosphorylation (Rojas et al., 2013).

Regrettably, there are only a few studies focusing on the regulation of iGluRs by group II and group III. For example, agonists of mGluR2 and mGluR3 can enhance NMDARs through a kinase-mediated pathway (Rosenberg et al., 2016). In the regulation of synaptic plasticity, iGluRs and mGluRs exhibit extensive antagonism and synergy. As mentioned above, group I mGluR mediates the enhancement of NMDAR, and the activation of group I mGluRs help increase the LTP amplitude in CA1 and CA3, whereby (the increase of LTP amplitude in CA1 is mainly influenced by mGluR5, and that in CA3 is influenced by mGluR1 (Berretta et al., 1993; Daniel et al., 1994; Lu et al., 1997). In addition, mGluR5 (Table 1), which is widely distributed in astrocytes, can not only promote the release of glutamate and apoptosis of astrocytes by activating and inducing IpA formation and intracellular Ca^2+^ increase (Miller et al., 1995; Pasti et al., 1997), but also inhibit microglia-related neuroinflammation by stimulating the MAPK pathway and PLD signaling (Servitja et al., 2001; Peavy and Conn, 2010).

The enhancement of mGluR-LTD in the hippocampus is strongly related to FXS, which will be described in detail in the disease-related part later. The mGluR-LTD depends on the increase of intracellular Ca^2+^ and the activation of postsynaptic group I mGluRs, especially the mGluR1 receptor (Alba et al., 1994; Shigemoto et al., 1994), rather than NMDAR. This is a key feature of mGluR-mediated LTD. However, NMDAR-LTD and mGluR-LTD are not mutually exclusive, and the induction mechanisms of these two LTD are inconsistent. Nevertheless, LTD in the perinasal cortex and amygdala requires the synergy of NMDAR and mGluRs for activation (Wang and Gean, 1999; Cho et al., 2000).

#### D-Serine

As mentioned above, NMDARs play an important role in excitatory nerve transmission and synaptic plasticity. However, the activation of NMDARs does not depend solely on glutamate, and requires D-ser or glycine as a co-agonist to bind to the GluN1 and GluN2 subunits, respectively. D-ser has also been thought to mediate some aspects of NMDAR-dependent neurodegeneration as the main NMDAR co-agonist in the forebrain (Billard, 2012).

As a typical glial transmitter, D-ser is synthesized by serine racemase (SR), which converts L-serine into D-serine. Selective deletion of SR can impair NMDAR-dependent synaptic plasticity (Benneyworth et al., 2012; Perez et al., 2017). Astrocytes supply the L-serine necessary for SR activity to neurons, which convert it into D-ser (Wolosker and Radzishevsky, 2013). Then, the D-ser synthesized by the neuron will be released to activate NMDAR (Hagit et al., 2016). Accordingly, the supply of L-serine by astrocytes promotes the synthesis of D-ser in neurons. As a co-agonist of NMDAR, D-ser then participates in the regulation of synaptic plasticity.

Ca^2+^-dependent D-ser release is also involved in the regulation of synaptic plasticity. Repeated synaptic activity increases the free Ca^2+^ of astrocytes and increases the production of D-ser in astrocytes in a short period of time. Ca^2+^-dependent D-ser release induces NMDAR-dependent LTP on excitatory synapses and controls the plasticity of thousands of nearby NMDAR-dependent excitatory synapses (Henneberger et al., 2010; Zhuang et al., 2011). Besides, the H1 receptor in the hippocampal CA1 region promotes the activity of NMDAR and induces the enhancement of EPSC and LTP in the synapses of neurons depending on the continuous activation of astrocytes and release of D-serine (Masuoka et al., 2019).

#### ATP

ATP can provide energy for cells as a direct energy material *in vivo*, and it is also one of the main diffusion signal molecules released by astrocytes. The ATP response in rodents is mediated by two P2 receptor families. The P2X receptor is a ligand-gated cationic channel that is permeable for Na^+^, K^+^, and Ca^2+^ (Burnstock and Kennedy, 2011; Saez-Orellana et al., 2015). ATP is the main endogenous agonist of all P2X receptors. P2Y receptors are G-protein-coupled receptors, which activate Ca^2+^ release by stimulating phospholipase C (PLC) (Mohapatra et al., 2011). The Ca^2+^ signal of human fetal astrocytes induced by ATP is completely mediated by P2Y1 and P2Y2 receptors, while P2X receptors do not affect it (Muller and Taylor, 2017; Lalo et al., 2019). When ATP is converted into adenosine, it can interact with A1 and A2A receptors to inhibit or enhance excitatory synaptic transmission. In the process of synaptic transmission, astrocytes increase the transmission efficiency of CA1 pyramidal cells by activating the subtype 5 metabotropic glutamate receptor (mGluR5) and presynaptic adenosine A2A receptor, resulting in the upregulation of synaptic transmission and induction of LTP (Panatier et al., 2011). The types of receptors activated by ATP affect different functions of astrocytes. For example, local Ca^2+^ responses are mediated by mGluR5, while other types of glutamate receptors such asNMDA receptors are involved in other forms of astrocyte excitation and excitatory synaptic plasticity (Halassa and Haydon, 2010).

### Extracellular Matrix

ECM molecules derived from astrocytes are also important factors in regulating synaptic function. In the central nervous system, the most prominent extracellular matrix structure is the peripheral neural network (PNN) (Lau et al., 2013). The PNN has the functions of producing polyanions that buffer the local environment, as well as capturing and locally concentrating growth factors and nutrients. Additionally, it can act as a diffusion barrier for extracellular signal molecules, including neurotransmitters in synapses, which helps prevent neurotransmitters from overflowing. Accordingly the PNN plays a role in stabilizing synapses, synaptic plasticity, and neuronal surface isolation (Suttkus et al., 2016; Sonntag et al., 2018).

Structurally, the PNN maintains the stability of chondroitin sulfate proteoglycans (CSPGs) binding to the hyaluronic acid (HA) skeleton by connecting proteins (Crtl1/Hapln1 and Bral2/Hapln4). In terms of composition, HA is the main component of the PNN, and CSPGs are its main proteoglycans. In the central nervous system, many CSGPs with different core proteins are secreted by astrocytes, such as brevican, versican and so on (Bandtlow and Zimmermann, 2000; John et al., 2006).

In EMC, the PNN is highly dynamic. After maturation, the PNN will establish a microenvironment between neurons and synapses, which will affect synaptic plasticity and determine where synapses form. The loosening of the PNN can destroy the stability of the inhibitory environment and release growth factors to promote synaptic plasticity (Dzyubenko et al., 2016).

When chondroitinase is injected into the central nervous system, it damages the PNN-like structure by destroying the chondroitin sulfate chain and hyaluronic acid (HA), which in turn impairs the transmission of excitatory synapses (Pyka et al., 2011). Therefore, PNN is a powerful factor in the effect of astrocytes on synaptic plasticity.

### Astrocyte Surface Proteins in Direct Contact With Synapses

Synapses are also affected by the direct adhesion between neurons and astrocytes. One factor mediating astrocyte-neuron adhesion is γ-Pcdh, and this astrocyte protein can promote the development of excitatory and inhibitory synapses (Garrett and Weiner, 2009; Pyka et al., 2011).

Studies have shown that the activation of protease-activated receptor 1 (PAR1) in hippocampal CA1 astrocytes leads to the opening of the Ca^2+^-dependent glutamate-permeable anion channel Best1, which mediates Ca^2+^-dependent glutamate release from astrocytes, thereby increasing the concentration of glutamate in the synaptic space (Woo et al., 2012). Synaptic NMDAR is the main target of Best1-mediated glutamate release in astrocytes. Increased activation of synaptic NMDAR leads to the enhancement of NMDAR-dependent synaptic transmission. When synaptic glutamate increases glutamate secretion in astrocytes mediated by Best1, the threshold of NMDAR-dependent LTP decreases (Park et al., 2015).

### Slow-Acting Astrocyte-Derived Cytokines

Astrocytes can secrete a variety of cytokines and neurotransmitters that participate in the regulation of synaptic plasticity. Hevin is a synaptic protein secreted by astrocytes. In the developing cortex, Hevin is necessary for maintaining synaptogenesis and maturation of the dendritic structure in the thalamic cortex (Risher et al., 2014). The transsynaptic adhesion between presynaptic neurexins (NRX) and postsynaptic neuroligins (NL) is essential for the formation and maturation of excitatory and inhibitory synapses (Baudouin and Scheiffele, 2010). Hevin can bridge Neuresin-1α and NeuroLigin-1B to assemble glutamatergic synapses. Thus, astrocytes change the cross-synaptic interaction between NLS and NRXs via Hevin, thereby regulating the formation and plasticity of excitatory synapses (Singh et al., 2016). Other synaptic proteins such as thrombospondin (TSP) play an important role in the formation of excitatory synapses (Chung et al., 2015; Baldwin and Eroglu, 2017). During development, the nervous system undergoes a process of forming a large number of neural circuits, during which the expression of TSP-1 and TSP-2 in astrocytes is maintained at a high level. Conversely, a lack of TSP will lead to a decrease in the number of excitatory synapses (Christopher et al., 2018). Glypicans can also increase the formation of excitatory synapses. Glypican4 and Glypican6 secreted by astrocytes can recruit AMPA glutamate receptors to the surface of postsynaptic cells and induce the formation of active excitatory synapses by increasing the density of receptors on the surface of postsynaptic cells (Allen et al., 2012; Baldwin and Eroglu, 2017; Farhy-Tselnicker et al., 2017). Transforming growth factor β1 (TGF-β1) is also secreted by astrocytes. As a member of the TGF-β superfamily, TGF-β1 has been recognized as a neuroprotective and neurotrophic factor in previous studies. Recent studies on the role of TGF-β1 in synaptic transmission have found that it is expressed in hippocampal neurons in an activity-dependent manner and participates in synaptic regulation (Caraci et al., 2015, 2018). *In vivo*, the levels of AMPA and NMDA receptor subunits increase in the hippocampi of mice with TGF-β1 overexpression, and the induction of excitatory synapses by TGF-β1 is mediated by NMDA receptor activity and the NMDA coactivator D-serine (Bae et al., 2011).

In addition, TGF-β1 can also induce a switch from the early stage of LTP, which is independent of protein synthesis (E-LTP) into the late stage that is dependent on protein synthesis (L-LTP), which indicates that TGF-β1 can increase neuronal excitability by inducing long-term dissimilation of ganglia (Caraci et al., 2015).

Brain-derived neurotrophic factor (BDNF) also plays an important role in regulating synaptic plasticity. BDNF works through two receptor systems, TrkB (tropomyosin-related kinase B) and P75NTR (Baldwin and Eroglu, 2017; Holt et al., 2019). Interestingly, the interaction of BDNF through p75NTR and Trk receptors often has opposite effects. For example, Trk receptor attachment almost always promotes neuronal survival and differentiation (Patel et al., 2000), whereas p75NTR involvement often promotes apoptosis (Defreitas et al., 2001). Similarly, in the case of synaptic plasticity, BDNF-mediated activation of TrkB promotes hippocampal LTP, whereas the interaction of neurotrophic factor with p75NTR instead promotes hippocampal LTD. A major pathway involved in p75NTR activation by neurotrophic factors is the Jun kinase signaling cascade, which leads to the activation of p53 and apoptosis. In addition, the Jun kinase cascade induces the expression of Fas ligands in neuronal cells, which promotes apoptosis by activating Fas receptors. There are important intermediates in the Jun kinase cascade pathway, such as NRIF and the E3 ubiquitin ligase Traf6. When these intermediates are absent, Jun kinase signaling in sympathetic neurons will also be blocked (Yeiser, 2004; Linggi et al., 2005). In PC12 cells and sympathetic neurons, the activation of the Jun kinase cascade involves Cdc42, and apoptosis is strongly inhibited by dominant-negative Cdc42 mutations (Bazenet and Rubin, 1998). Binding of neurotrophic factor to p75NTR also promotes NF-κB activation, thereby promoting NF-κB-dependent neuronal survival (Middleton, 2000). The involvement of neurotrophic factors in p75NTR also controls Rho family GTPase activity. It was shown that p75NTR activates RhoA through direct interaction, thereby inhibiting neurite growth. The binding of neurotrophic factor to p75NTR eliminates p75NTR-dependent RhoA activation and stimulates neurite growth (Yamashita et al., 1999). All of these studies illustrate the self-evident importance of p75NTR as a death receptor in neural and synaptic development.

**Figure 2:**
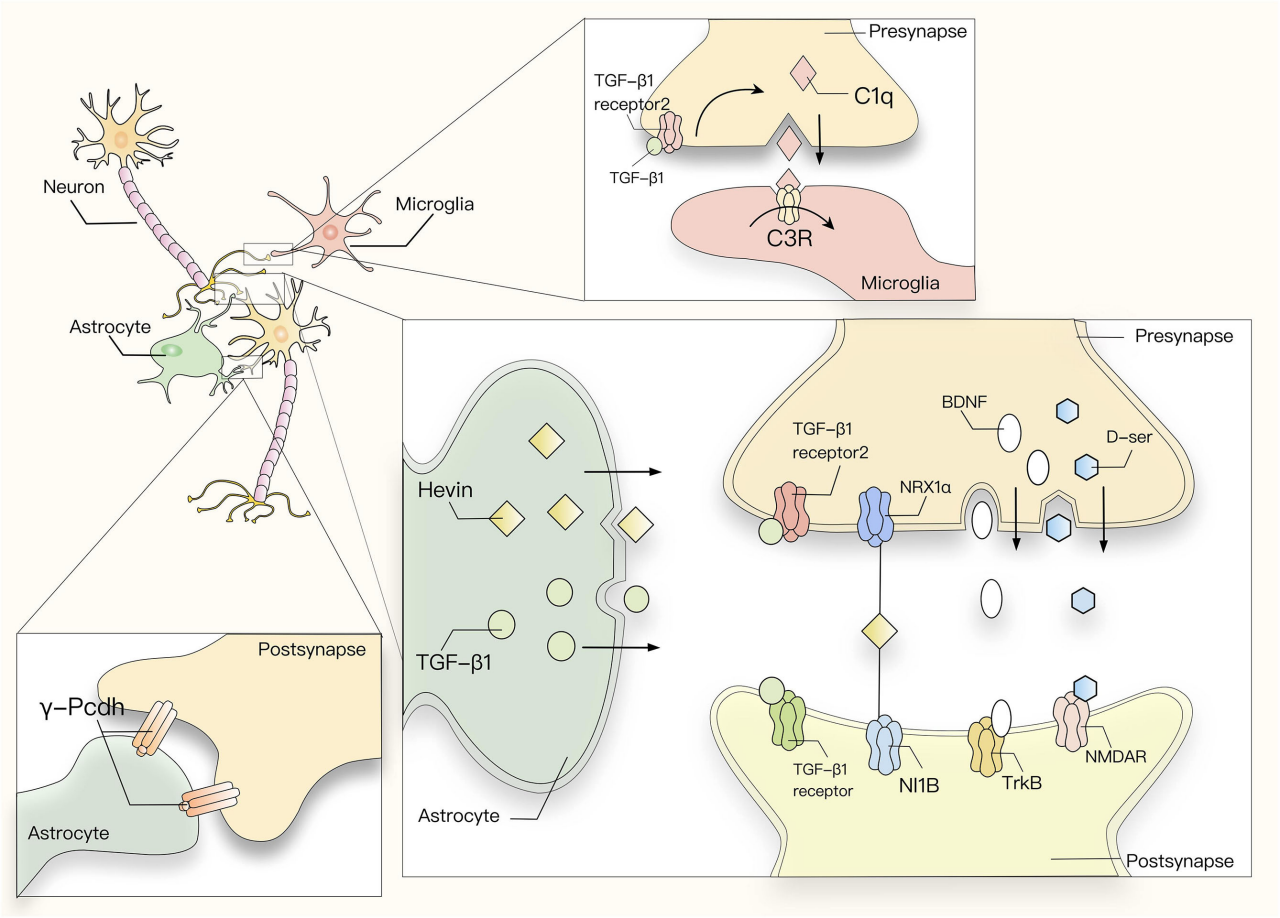
Astrocytic cytokines and neurotransmitters participate in the regulation of synaptic plasticity. TGF-β1 secreted by astrocytes induces the formation of inhibitory synapses by activating neuronal calcium/calmodulin dependent protein kinase II (CaMK II), or promotes the formation of excitatory synapses through an activity-dependent mechanism of the NMDA receptor together with its agonist D-ser. In addition, the development of both excitatory and inhibitory synapses is affected by astrocyte-neuron adhesion mediated by astrocytic γ-Pcdh. Astrocytes secrete Hevin to interact with two proteins, presynaptic NRX1α and postsynaptic NL1b, which usually do not interact with each other, thus promoting synapse formation. On the other hand, BDNF regulates LTP mainly by binding to the TrkB receptor. Astrocytes can also induce the production of complement protein C1q by secreting TGF-β and thereby acting on TGFBR2 in the synapse. C1q is recognized by microglia and triggers C3R-mediated phagocytosis.

In terms of synaptic plasticity, the effect on LTP is mainly mediated by the TrkB receptor. Stable LTP in rodent DG requires a BDNF-TrkB signal period lasting more than 8 h after LTP induction (Panja et al., 2014). Unprocessed BDNF is also called proBDNF, and its synaptic effect is opposite to that of mature BDNF, which acts through TrkB. By contrast, proBDNF has a high affinity for the p75NTR (Chao and Bothwell, 2002; Lu, 2003). ProBDNF binds to P75NTR first, and their binding induces LTD (Rösch et al., 2005). In the normal physiological state, the expression of P75NTR in the nervous system is very limited, and it is upregulated in pathological conditions such as inflammation or seizures. Therefore, the influence of BDNF on LTD is minimal (Figure 2). Studies have also shown that cholecystokinin (CCK) and glutamate cooperate with the CCK receptor and mGluR5 in astrocytes to trigger the release of the glial transmitter ATP, which increases the release of GABA from adjacent inhibitory terminal synapses, and switches the state of GABAergic synapses in the dorsomedial hypothalamic nucleus of rats from LTD to LTP (Crosby et al., 2018).

## Roles of Astrocytic Factors in the Regulation of Inhibitory Synaptic Function

### Astrocytic Exocrine1 Factors

GABA is the main inhibitory neurotransmitter, and astrocytes express the GABA transporters GAT1 and GAT3 (Scimemi, 2014). Changes in the expression and activity of GAT1 or GAT3 affect inhibitory synaptic transmission in inter-hippocampal neurons (Beenhakker and Huguenard, 2010). GATs control the excitability of neurons in the neural network by regulating the level of extracellular GABA (Muthukumar et al., 2014). Furthermore, there is a signaling pathway between astrocytes and the GABA system. Astrocytes can enhance the current mediated by GABA in neurons and increase the transmission of inhibitory synapses in hippocampal CA1 neurons through Ca^2+^ signals. GABA selectively opens the Cl-channel by activating the type A GABA receptor (GABAAR), which induces an inhibitory potential and mediates rapid synaptic inhibition. The activation of GABAb elevates the Ca2+ level, which at the same time enhances the Ca2+-dependent release of gliotransmitters from astrocytes (Kang et al., 1998). It is now generally accepted that synaptic inhibition is mediated by GABAA receptors and that potentiation involves astrocyte GABAB receptors, astrocyte glutamate release, and presynaptic metabotropic glutamate receptors. And interneuron activity regulates the release of transmitters from the same synapses according to the firing rate, which suggesting that mechanisms of synaptic inhibition can coexist at the same synapse as those responsible for synaptic potentiation and that the end result is regulated by the firing rate of the interneuron (Perea et al., 2016). In a mouse model of Alzheimer's disease (AD), the excess GABA released by reactive astrocytes through the Best1 channel in the hippocampus acts on presynaptic GABA receptors and leads to catatonic inhibition of the dentate gyrus granulosa cells in the hippocampus (Lee et al., 2010; Jo et al., 2014). In terms of memory impairment caused by AD, inhibition of GABA produced by reactive astrocytes or blocking of GATs by different means can restore synaptic plasticity, learning, and memory impairment caused by AD. The application of GABA receptor antagonists in AD mice can improve hippocampal LTP, alleviate the learning impairment, and protect memory to different degrees (Yuji et al., 2008). Therefore, it stands to reason that inhibition of the synthesis or release of GABA may be a powerful method for treating memory impairment caused by AD.

TGF-β1 not only promotes the formation of excitatory protrusions, but also promotes the formation of inhibitory synapses. It induces the formation of inhibitory synapses by increasing the phosphorylation of CaMKII downstream of NMDA receptors, as well as increasing the expression of NL2 and aggregation of Gephyrin/NL 2, which is an important component of inhibitory synapses (Diniz et al., 2015).

At present, it is believed that Chordin-like1 (Chrdl1) factor secreted by astrocytes can regulate the level of synaptic GluA2AMPAR, mainly by increasing its quantity. In the developing brain, an important sign of the stability and maturation of excitatory glutamatergic synapses is that AMPAR subtypes on excitatory synapses are replaced by Ca^2+^-opaque channels containing GluA2 from Ca^2+^ permeable channels (Brill and Huguenard, 2008). In other words, by reducing the entry of calcium into postsynaptic cells, excitatory glutamatergic synapses tend to mature, stabilize, and limit synaptic plasticity mediated by Ca^2+^-dependent pathways (Henley and Wilkinson, 2016). However, the mechanism of the iconic transformation of stable mature synaptic AMPAR is not clear. Studies show that synaptic GluA2 AMPAR decreases and synaptic remodeling increases in response to changing sensory inputs in Chrdl1KO mice. In other words, Chrdl1 expressed by astrocytes limits synaptic plasticity by promoting GluA2-dependent synaptic maturation (Henley and Wilkinson, 2016).

With regard to the effect of ATP on LTD, researchers found that selective stimulation of astrocytes through ChR2 could lead to LTD, while blocking P2Y receptors in neighboring hippocampal neurons could prevent hLTD. Thus, ATP secreted by astrocytes mediates hippocampal LTD by activating neuronal P2Y1 receptors (Chen et al., 2013).

### Other Factors

Recent studies have shown that the number and transmission of inhibitory synapses in neurons co-cultured with astrocytes is increased compared with neurons cultured alone, and their plasticity is enhanced (Sorg et al., 2016). Interestingly, these effects disappeared after treatment with selective Krebs cycle inhibitors such as fluoroacetic acid, indicating that the regulation of GABAergic synaptic development depends on a series of metabolic pathways in astrocytes, including the Krebs cycle. In other words, key metabolic enzymes expressed by astrocytes, such as glutamine synthetase, contribute to the plasticity of GABAergic neuronal networks (Kaczor and Mozrzymas, 2017).

## Pruning Effect of Astrocytes on Synaptic Morphology

In order to form functional neural circuits, the initially formed excess synapses are removed during brain development. This kind of pruning plays an important role in normal synaptic development and neural circuit formation in the CNS. Recent studies have found that astrocytes are involved in this synaptic pruning process.

### Direct Effects

**Figure 3:**
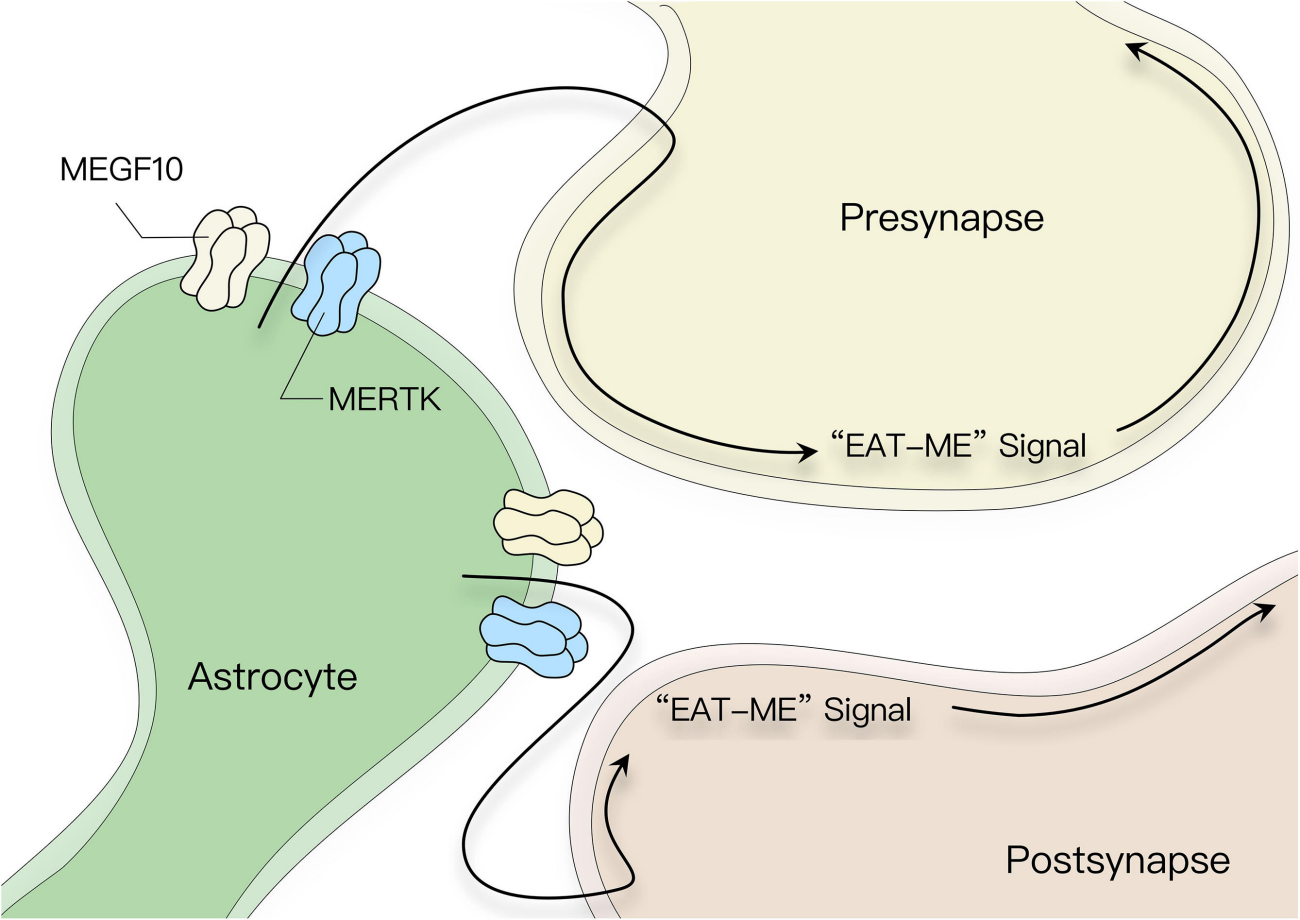
Pruning effect of astrocytes on synaptic morphology. Astrocyte surface receptors MEGF10 and MerTK recognize phosphatidylserine in the target fragment as an opsonic signal, which induces astrocytes to eliminate superfluous synapses in the CNS.

Synaptic phagocytic receptors MEGF10 and MerTK are expressed on the surface of astrocytes. Astrocytes can eliminate excitatory or inhibitory synapses by interacting with MEGF10 and MerTK, which can recognize phosphatidylserine in target fragments as an opsonic signal to initiate phagocytosis and drive synaptic remodeling (Figure 3) (Chung et al., 2013). This process of synaptic elimination is strongly dependent on neural activity. Recent studies have shown that astrocytes regulate synaptic elimination by activating purinergic signals in an ATP-dependent manner mediated by IP3R2 receptor release (Yang et al., 2016).

In addition, studies have shown that astrocytes can express Ephrin-B1, which is a membrane binding protein and the ligand of the EphB receptor. The cross-synaptic ephrin-B/EphB interaction between neurons is essential for the formation and maintenance of synapses in the mouse brain (Henkemeyer et al., 2003; Grunwald et al., 2004; Xu and Henkemeyer, 2012). Astrocytic ephrin-B1 can compete with neuronal ephrin-B1 and trigger astrocyte-mediated elimination of synapses containing EphB receptors through cross-synaptic endocytosis. The loss of the neuronal EphB receptor weakens the ability of astrocytes expressing functional Ephrin-B1 to phagocytize synaptosomes *in vitro*, while the overexpression of Ephrin-B1 in astrocytes impairs long-term contextual memory. Thus, astrocytic Ephin-B1 regulates long-term contextual memory by limiting the formation of new synapses in the adult hippocampus (Koeppen et al., 2018).

Astrocytes are the main source of apolipoprotein E (ApoE) in the central nervous system and transport cholesterol to neurons through apolipoprotein E receptors, a member of the (LDLR) family of low-density lipoprotein receptors. ApoE exists in three subtypes, ApoE2, ApoE3, and ApoE4, whereby the latter is related to the decrease of dendritic spine density in memory retention (Caselli et al., 2004). The formation of dendritic spines is the most important feature of synaptic plasticity of mature neurons. At the same time, AopE4 is also the strongest genetic risk factor for late-onset Alzheimer's disease (LOAD).

### Indirect Effects

Astrocytes can also initiate the classical complement cascade by secreting TGF-β and inducing the expression of complement protein C1q *via* the TGFBR2 receptor. C1q is then recognized and taken up by microglia through C3R-mediated phagocytosis (Schafer et al., 2012; Bialas and Stevens, 2013). In this way, astrocyte-derived TGF-β regulates the expression of neuronal C1q to initiate complement activation and microglial-mediated synaptic pruning (Figure 2).

In addition to complement C1q, IL-33 expressed by astrocytes also drives microglial synaptic phagocytosis and limits the number of excitatory synapses (Nguyen et al., 2020). These studies have shown that astrocytes can mediate the indirect effects of microglia-dependent synaptic elimination through C1q or IL-33.

## Implications of Astrocyte Cell Biology for Human Diseases

Astrocytes play important roles in a range of neurodevelopmental and psychiatric disorders in humans, and a better understanding of the function of astrocytes is needed to understand the pathological changes that lead to these disorders. Because astrocytes are so closely related to synapses and influence synapse formation, maturation and plasticity, their dysfunction may be closely associated with psychiatric disorders such as schizophrenia, autism and depression. In addition, astrocyte dysfunction has also been implicated in the pathology of human neurodevelopmental disorders such as Rett syndrome, fragile X syndrome, and Down syndrome.

### Astrocytes and Autism Spectrum Disorders

Autism spectrum disorders (ASD) are a heterogeneous group of neurological developmental conditions characterized by repetitive movements, impairments in social interaction, and altered vocal communication as the core symptoms (Lai et al., 2014). The exact pathophysiological basis of ASD is currently unknown, but a growing body of evidence supports dysregulated neuroinflammation and dynamic imbalances in synapse formation (Christopherson et al., 2005; Kucukdereli et al., 2011), pruning, elimination (Chung et al., 2013), and plasticity (Henneberger et al., 2010) as important factors contributing to ASD. Neuroinflammation is characterized by the persistent activity and proliferation of glial cells (e.g., astrocytes and microglia) following injury, infection, or disease. Astrocytes respond to neuroinflammation through morphological changes and the release of pro-inflammatory mediators (Hanisch and Kettenmann, 2007; Kierdorf and Prinz, 2013). Since astrocytes are closely associated with the connection and regulation of neural circuits, early disruption of astroglial homeostasis may lead to abnormal brain development and the ASD phenotype. Autopsy studies have found that individuals with ASD have increased numbers of neurons in the cerebral cortex, white matter, and especially the cerebellum (Morgan et al., 2010; Vargas et al., 2010). Western blot analysis indicated that GFAP protein expression was significantly increased in the cerebellum, frontal middle gyrus, and anterior cingulate gyrus of autistic patients, suggesting that astrocyte immunoreactivity was also increased (Vargas et al., 2010). Recent studies have shown that astrocytes control excitatory synaptogenesis through the secretion of platelet responsive protein (TSP), which acts through its neuronal receptor calcium channel subunit α2δ-1 (Eroglu et al., 2009). The α2δ-1 receptor is associated with neurological disorders such as epilepsy, neuropathic pain, intellectual disability and ASD (Newton et al., 2001; Iossifov et al., 2014). The small Rho GTPase Ras-Related C3 Botulinum toxin substrate 1 (RAC1) is a synaptic signaling protein downstream of the TSP-α2δ-1 axis. RAC1 is a key component of the cascade that mediates synaptic and spinal growth (Christopher et al., 2018). This provides a new perspective for intervention in diseases including ASD and epilepsy, which are strongly associated with abnormal RAC1 signaling (Zeidán-Chuliá et al., 2013; Tejada-Simon, 2015). Since astrocytes can control neuronal RAC1 signaling through TSP-α2δ-1, understanding the regulatory mechanism of the TSP-α2δ-1-RAC1 pathway in astrocytes may uncover new potential therapeutic targets for psychiatric disorders such as ASD in the future.

### Astrocytes and Rett Syndrome

Rett syndrome (RTT) is a rare X-linked neurodevelopmental disorder resulting in a range of symptoms including autistic features, intellectual impairment, motor degeneration and autonomous nervous system abnormalities. Loss of function of the X-linked gene MECP2, which encodes methyl CpG-binding protein 2, is the main cause of RTT (Amir et al., 1999). MECP2 is both a transcriptional activator and a transcriptional repressor that regulates synaptic activity in an activity-dependent manner (Qiu et al., 2012). Although few early studies explored the role of glial cells in RTT, advances in technology have shown that MECP2 is expressed in both astrocytes and neurons (Grunseich et al., 2009). In fact, a recent study found that naïve dendrites of primary WT neurons co-cultured with MECP2-deficient primary astrocytes were poorly developed compared to neurons co-cultured with WT primary astrocytes (Grunseich et al., 2009; Maezawa et al., 2009). Moreover, dendritic and synaptic morphology could be restored *in vivo* after restoring MECP2 (Lioy et al., 2011). These findings that MECP2-deficient astrocytes are detrimental to neuronal and dendritic growth and development, and that astrocytic MECP2 deficiency is an important cause of RTT. This morphological abnormality of neurons and dendrites has been attributed to neurotoxins released by MECP2-deficient astrocytes. However, how the astrocytes of RTT patients exert neurotoxic effects remains unknown. Furthermore, the transcription of the neurotrophic factor BDNF secreted by astrocytes is also regulated by MECP2, and overexpression of BDNF in MECP2-deficient mice improved motor function and restored the normal electrophysiological activity of somatosensory pyramidal neurons (Chang et al., 2006). Based on this, we can ask the question whether MECP2 can also control other factors secreted by astrocytes (such as TSPs, Hevin, etc.).

### Astrocytes and Fragile X Syndrome

Among the many forms of inherited intellectual disability, Fragile X syndrome (FXS) is one of the most common. The trinucleotide repeat amplification of the Fragile X mental retardation 1 (FMR1) gene can lead to the loss of Fragile X mental retardation 1 protein (FMRP), which plays a number of important roles in the human body, such as binding to proteins involved in the regulation of genomic stability, cell differentiation and other processes (Contractor et al., 2015). It also plays an important role in the translation, transport and targeting of neuronal mRNAs, which in part control synaptic plasticity, development and elimination (Pfeiffer and Huber, 2009). FXS is mainly characterized by moderate to severe mental retardation, autistic traits, susceptibility to seizures, and behavioral abnormalities (Penagarikano, 2007). FMR1 knockout (KO) mice have phenotypes such as learning and intellectual deficits (Comery et al., 1997; Wisniewski et al., 2010), as well as changes of social behavior (Mckinney et al., 2010) that are similar to those of human FXS patients.

*In vivo* imaging studies on global FMR1 KO mice indicated that the lack of FMRP leads to reduced synaptic stability (Feng et al., 2010; Padmashri et al., 2011). In addition, the density of immature dendritic spines in cortical vertebral neurons is greatly increased in both FXS patients and adult FMR1 KO mice (Dailey and Smith, 1996). The morphology and density of dendritic spines has important implications for proper synaptic connectivity and function (Sala and Segal, 2014). Therefore, the lack of FMRP will lead to abnormal development of dendritic spines and thus affect synaptic connections and functions. For example patients with fragile X syndrome, the spine is usually longer and thinner than the average person and there are more spines exist per unit length of the dendrite (Irwin et al., 2001), which lead to a series of abnormalities social behavior such as verbal language deficits, reduced eye contact, social and generalized anxiety, sensory hypersensitivity, and difficulty regulating attention and activity levels (Lewis et al., 2010). These abnormalities in social behavior will continue from the onset into adulthood. Also, Patients with fragile X syndrome have a learning disability, as both learning and memory are impaired in patients with fragile X syndrome. A recent study showed that selective deletion of the FMR1 gene in astrocytes results in reduced expression of the astroglial glutamate transporter protein GLT1 (Higashimori et al., 2016). The primary role of GLT1 is the regulation of the extracellular synaptic glutamate concentration, and GLT-1-mediated loss of glutamate uptake increases extracellular glutamate levels, thereby increasing excitability of layer V pyramidal neurons (Higashimori et al., 2016). The increased glutamate concentration may promote the growth of new dendritic spines (Kwon and Sabatini, 2011). Thus, the dysregulation of glutamate homeostasis caused by the absence of FMRP in astrocytes may partially explain the increased density of immature dendritic spines in FMR1 KO mice. Astrocyte-specific deletion of FMR1 leads to a significant increase in immature spines in the motor cortex of mice, which manifests as abnormal synaptic morphology and reduced synaptic density in hippocampal neurons. This is due to the overproduction of dendrites during neuronal development and abnormal or insufficient pruning (Hodges et al., 2016). Interestingly, normal astrocytes prevent such abnormal synapses and reduced density of hippocampal neurons. Therefore, astrocytes play an important role in the abnormal synaptic development of FMR1 KO mice.

The group I mGluRs (mGluR1/5)-mediated LTD is a specific protein synthesis-dependent synaptic plasticity, which can be increased in FMR1KO mice (Huber et al., 2002). This groundbreaking discovery led to a new mGluR-based theory of FXS, which states that dysregulated mGluR1/5-mediated protein synthesis-dependent forms of synaptic plasticity contribute to the pathology of FXS (Bear et al., 2004). The synthesis of this specific protein leads to the internalization of AMPAR, which is the key mechanism of mGluR-LTD. Unfortunately, this mechanism seems to be damaged in FXS. Some studies have found that the mGluR5-mediated internalization of AMPAR increases when FMRP levels decrease (Nakamoto et al., 2007). Therefore, we have reason to believe that the role of FMRP in regulating mGluR1/5-induced protein synthesis may be the basis of LTD, exemplified in FXS mice. In addition, the sensitivity of mGluR1/5 to glutamate released by synapses in the hippocampus is enhanced in FXS mice, and the resulting loss of FMRP also leads to prolonged epileptiform discharges (Chuang, 2005; Bianchi et al., 2009). Moreover, the synthesis and release of endogenous cannabinoids in the hippocampus cannabinoid (eCB) mediated by mGluR1/5 is in enhanced following the deletion of FMRP (Zhang and Alger, 2010). In fact, there are also defects of the mGluR1/5-dependent pathway in other areas of the FXS mouse brain, such as abnormal synaptic plasticity of mGluR1/5-dependent neurons in the amygdala of FMR1 KO mouse (Suvrathan et al., 2010). Similar to the excessive LTD in the hippocampus, there is also excessive mGluR1/5-dependent LTD in the cerebellum of FXS mice (Koekkoek et al., 2005). Since different areas of the brain correspond to different functions, defects of the mGluR1/5-dependent pathway in different areas of the FXS brain may not only lead to cognitive dysfunction, but also lead to epilepsy (prolonged epileptiform discharges in the hippocampus), anxiety (abnormal synaptic plasticity in the amygdala) and motor deficits deficit (cerebellum). Although most of these studies are based on FXS animal models that may not always completely reflect the human phenotype, the successful validation of mGluR theory in different animal models further confirmed that FMRP plays a vital role in stimulation-induced mGluR1/5 signal transduction, and the potential value of glutamate derived from astrocytes via to activate mGluR1/5 as a therapeutic target for the treatment of FXS.

### Astrocytes and Down Syndrome

Trisomy of chromosome 21 causes Down syndrome (DS), which is the most common cause of inherited mental retardation. Intellectual disability in DS includes deficits in cognitive function, interpersonal interaction and communication. Abnormalities in the structure and function of dendritic spines are one of the prominent features of DS. Altered dendritic spine morphology and reduced density may underlie altered neuronal and synaptic plasticity (Benavides-Piccione et al., 2004), ultimately leading to cognitive deficits. In the cortical and hippocampal structures of DS fetuses and neonates, the morphology of dendritic spines exhibits abnormal changes, such as unusually long spines, shorter spines, and a decrease in synaptic density (Marin-Padilla, 1972). It was found that this abnormal dendritic spine development as well as the reduced synaptic density and activity in DS patients is closely related to astrocytes, and that the astrocyte-secreted TSP-1 protein is a key factor that regulates the number and morphology of dendritic spines. TSP-1 levels are significantly reduced in the astrocytes of DS patients, and restoration of TSP-1 levels can rescue the aforementioned morphological abnormalities and synaptic defects in the dendritic structures of hippocampal and cortical neurons (Octavio et al., 2010). Such findings highlight the role of astrocytes in synaptic defects in DS, and although there are no effective methods to date to prevent or restore such synaptic defects, these results demonstrate the potential of astrocyte-secreted molecules such as TSP-1 as therapeutic targets for symptomatic relief in DS and other neurological disorders associated with synaptic defects.

Indeed, astrocytes may influence numerous neurological disorders in multiple ways, and it is well-known that targeting neurons will be far more difficult than targeting astrocytes, which speaks volumes about the potential of astrocytes as therapeutic targets for the prevention and treatment of related diseases.

## Conclusions

The concept of a “tripartite synapse” allows us to re-examine the role of astrocytes in neural circuits, rather than simply treating them as supporting cells that passively fill the gaps between neurons. As a component of the synapse, astrocytes promote synaptogenesis and regulate synaptic connectivity by influencing synapse formation, elimination, and maturation by secreting proteins, lipids, and small molecules that bind to neuronal receptors. Synaptic dysfunction is a prominent feature of various neurological diseases, including Alzheimer's disease, autism, and schizophrenia. Therefore, the importance of astrocytes is self-evident. Astrocytes secrete neurotransmitters such as glutamate and ATP that affect intracellular Ca^2+^ levels by acting on corresponding metabolic receptors. Astrocytes also initiate and maintain Ca^2+^ waves between neurons and astrocytes, and induce LTP. We discussed that the increase of Ca^2+^ levels in astrocytes has a direct effect on the release of synaptic neurotransmitters and synaptic plasticity, but the research on the effects of astrocytic signal characteristics on the nervous system, such as the rate of Ca^2+^ increase in astrocytes, the rise in subcellular regions or the mechanism underlying the effects of IP3-dependent and -independent signals on the nervous system and astrocytes is still insufficient. At the same time, how the diverse glial transmitters are released from astrocytes under the influence of Ca^2+^, the release pathway, and the mechanism are still controversial. If future research can clarify these problems, it will greatly promote our understanding of how astrocytes affect synaptic plasticity.

In addition, most studies have focused on the role of astrocytes in regulating the formation, function or elimination of excitatory synapses, while little is known about the role of inhibitory synapses or circuits. Only a few studies have reported the mechanism through which astrocytes influence the development of GABAergic synapses and related neural circuits. At the same time, many studies have begun to explain the relationship between astrocytes and neurodegenerative diseases.

The connection of targeted neurons is much more difficult than that of targeted glial cells, so astrocytes are a better intervention target than neurons during growth and development. The study of the influence of astrocytes on synaptic plasticity in the development of neural circuits can not only enhance our understanding of healthy synaptic development, but also guide the search for new treatments against neurodegenerative diseases.

## Author Contributions

XL and FH contributed in study design, literature search, and manuscript preparation. JY and XW helped conceptualize, write the original draft, and review and edit the manuscript. QZ, TZ, SY, WY, DY, and YF helped review and edit the manuscript. This manuscript was handled by XL. All authors contributed to the article and approved the submitted version.

## Funding

This work was supported by grants from the National Natural Science Foundation of China (81760261 and 82060219); Provincal Science foudation of Jiangxi (20192BCB23024 and 20202BABL206016); Youth Team Project of the Second Affiliated Hospital of Nanchang University (2019YNTD12003).

## Conflict of Interest

The authors declare that the research was conducted in the absence of any commercial or financial relationships that could be construed as a potential conflict of interest.

## Publisher's Note

All claims expressed in this article are solely those of the authors and do not necessarily represent those of their affiliated organizations, or those of the publisher, the editors and the reviewers. Any product that may be evaluated in this article, or claim that may be made by its manufacturer, is not guaranteed or endorsed by the publisher.
